# A Haloarchaeal Small Regulatory RNA (sRNA) Is Essential for Rapid Adaptation to Phosphate Starvation Conditions

**DOI:** 10.3389/fmicb.2019.01219

**Published:** 2019-06-05

**Authors:** Jana Kliemt, Katharina Jaschinski, Jörg Soppa

**Affiliations:** Biocentre, Institute for Molecular Biosciences, Goethe University Frankfurt, Frankfurt, Germany

**Keywords:** archaea, *Haloferax volcanii*, small regulatory RNA, phosphate starvation, ABC transporter, transient regulation, sRNA regulon

## Abstract

The haloarchaeon *Haloferax volcanii* contains nearly 2800 small non-coding RNAs (sRNAs). One intergenic sRNA, sRNA132, was chosen for a detailed characterization. A deletion mutant had a growth defect and thus underscored the importance of sRNA132. A microarray analysis identified the transcript of an operon for a phosphate-specific ABC transporter as a putative target of sRNA132. Both the sRNA132 and the operon transcript accumulated under low phosphate concentrations, indicating a positive regulatory role of sRNA132. A kinetic analysis revealed that sRNA132 is essential shortly after the onset of phosphate starvation, while other regulatory processes take over after several hours. Comparison of the transcriptomes of wild-type and the sRNA132 gene deletion mutant 30 min after the onset of phosphate starvation revealed that sRNA132 controls a regulon of about 40 genes. Remarkably, the regulon included a second operon for a phosphate-specific ABC transporter, which also depended on sRNA132 for rapid induction in the absence of phosphate. Competitive growth experiments of the wild-type and ABC transporter operon deletion mutants underscored the importance of both transporters for growth at low phosphate concentrations. Northern blot analyses of four additional members of the sRNA132 regulon verified that all four transcripts depended on sRNA132 for rapid regulation after the onset of phosphate starvation. Importantly, this is the first example for the transient importance of a sRNA for any archaeal and bacterial species. In addition, this study unraveled the first sRNA regulon for haloarchaea.

## Introduction

Small non-coding regulatory RNAs (sRNAs) have been found in all three domains of life. Eukaryotes contain thousands of very small sRNAs of about 20 nt (miRNAs, siRNAs, piRNAs) as well as longer sRNAs of up to several hundred nucleotides (lncRNAs). Eukaryotic sRNAs are involved in various processes, and their mis-regulation can lead to various diseases as well as cancer (Hombach and Kretz, 2016; Rajan et al., 2017; Romano et al., 2017; Subhramanyam and Hu, 2017).

sRNAs have also been identified in all archaeal and bacterial species that were investigated. They are typically between 100 nt and 500 nt long and can be divided into three classes,i.e., intergenic sRNAs (igRNAs), *cis* antisense sRNAs (asRNAs), and *cis* sense RNAs (isRNAs). sRNAs can be involved in many different biological functions, e.g., metabolic regulation, stress adaptation, biofilm formation, and virulence. Several reviews give overviews on the sRNA repertoire and sRNA functions in bacteria (Waters and Storz, 2009; Georg and Hess, 2011; Kopf and Hess, 2015; Murina and Nikulin, 2015; van Puyvelde et al., 2015; Wagner and Romby, 2015; Georg and Hess, 2018) and in archaea (Babski et al., 2014; Wagner and Romby, 2015; Kliemt and Soppa, 2017; Gelsinger and DiRuggiero, 2018a; Prasse and Schmitz, 2018).

Highly parallel transcriptome analyses with RNA-Seq or dRNA-Seq have revealed that in bacteria total numbers of sRNAs as well as their distribution to the three classes is species-specific and can vary widely, e.g., from less than 300 for *Salmonella* (Ramachandran et al., 2014) to more than 8000 for *Anabaena* spec. PCC 7120 (Kopf and Hess, 2015). RNA-Seq and dRNA-Seq studies have also been performed with a limited number or archaeal species, i.e., *Haloferax volcanii* (Babski et al., 2016; Gelsinger and DiRuggiero, 2018b; Laass et al., 2019), *Methanolobus psychrophilus* (Li et al., 2015), *Methanosarcina mazei* Gö1 (Jäger et al., 2009), *Pyrococcus abyssi* (Toffano-Nioche et al., 2013), *Sulfolobus solfataricus* (Wurtzel et al., 2010), and *Thermococcus kodakarensis* (Jäger et al., 2014). Again, the numbers were very different, e.g., only about 300 sRNAs were reported for *S. solfataricus*, while 2800 non-coding RNAs were found for *H. volcanii*.

*H. volcanii* is a model archaeon that is well suited for molecular genetic analyses (Soppa et al., 2008). The first sRNAs of *H. volcanii* have been discovered nearly 10 years ago using small scale RNomics (Straub et al., 2009). Since then, bioinformatics predictions, mixed RNA-Seq, dRNA-Seq, and comparative dRNA-Seq were used to characterize its sRNA repertoire, the length distribution of the different classes of sRNAs, as well as differential expression (Babski et al., 2011, 2016; Heyer et al., 2012; Gelsinger and DiRuggiero, 2018b; Laass et al., 2019). In addition, 28 sRNA gene deletion mutants have been generated to unravel putative biological functions. For the majority of mutants phenotypic differences to the wildtype could be found, and the phenotypes indicated that sRNAs are important for many biological functions in *H. volcanii*, including metabolic regulation, stress adaptation, and swarming (Jaschinski et al., 2014).

However, while the genetic approach was informative for many sRNAs, various sRNA deletion mutants behaved very similar or identical to the wildtype under all ten conditions tested (Jantzer et al., 2011). Therefore, we have chosen one of these sRNAs, sRNA132, to apply alternative approaches for the elucidation of its function. The transcriptomes of the sRNA132 deletion mutant and the wildtype were compared with a DNA microarray, which yielded a first indication to a target mRNA. Extensive Northern blot analyses and additional DNA microarray analyses enabled to unravel the sRNA132 regulon and showed that it is important for the transition to starvation conditions. Taken together, this is the first molecular analysis of a haloarchaeal sRNA and its biological function.

## Materials and Methods

### Strains, Media, and Growth Conditions

The *H. volcanii* strain H26 was used as a parent strain and wildtype for this study (Allers et al., 2004). It is a *pyrE2* deletion mutant, which enables the easy generation of deletion mutants. The deletion mutant of the sRNA132 gene has been described before (Jaschinski et al., 2014), generation of the remaining deletion mutants is described below.

The *H. volcanii* strains were grown in complex medium with the optimal NaCl concentration of 2.2 M as described (Jantzer et al., 2011). In short, 30 ml cultures were grown in 100 ml Erlenmeyer flasks at 42°C with good aeration (250 rpm). Growth was monitored spectroscopically at 600 nm or with a counting chamber.

The *Escherichia coli* strain XL1-blue MRF’ (Agilent Technologies, Waldbronn, Germany) was used for cloning, it was grown under standard conditions (Green and Sambrook, 2012).

### Generation of Deletion Mutants

For the generation of deletion mutants, the so called Pop-In-Pop-Out method was used (Allers et al., 2004). In short, two PCR fragments of about 500 nt were generated, which represent genomic regions upstream and downstream of the desired deletion. The PCR fragments were fused via a third PCR reaction and cloned into the suicide vector pMH101 (Hammelmann and Soppa, 2008). The resulting vector was used to transform *H. volcanii* H26, and the integration of the plasmid via homologous recombination was selected by growth in the absence of uracil. The selection for plasmid loss via a second homologous recombination event was selected by growth in the presence of uracil and 5-fluoroorotic acid, which results in the formation of the deletion mutant and the wildtype with equal probability. Colony PCR was used to identify deletion mutants, which were further characterized by Southern blot analysis. In this study, multigene deletions of the operons HVO_A0477 to HVO_A0480 and HVO_2375 to HVO_2378 as well as a double deletion mutant of both operons have been generated. The primers to generate the PCR fragments for the deletions and the probes for Southern blot analyses are summarized in Supplementary Table S1.

### Competitive Growth Analysis

For competitive growth analyses, the gene for the phytoene dehydrogenase (HVO_2528) was deleted from the genome of H26, resulting in a strain that was devoid of carotinoid biosynthesis and thus formed white colonies (Maurer et al., 2018). Cultures of the two operon deletion mutants (see above), which formed red colonies, and the HVO_2528 deletion mutant were grown to mid-exponential growth phase (about 4 × 10^8^ cells ml^-1^). 5 × 10^6^ cells from the HVO_2528 deletion mutant and the respective operon deletion mutant were used to inoculate fresh medium, which contained the reduced phosphate concentration of 10 μM (HVO_A0477-80) or 50 μM (HVO_2375-78) instead of the normal concentration of 1 mM. The culture was grown overnight, and an aliquot of 1 × 10^7^ cells was used to inoculate fresh medium with the same reduced phosphate concentration. Another aliquot was used to generate serial dilutions, which were plated and used to quantify the fractions of red and white colonies. This procedure was repeated several days, as indicated in Figure 7.

### Northern Blot Analysis

Cultures were grown to mid-exponential growth phase as described above. RNA isolation, probe generation, and Northern blot analyses were performed as described previously (Herrmann and Soppa, 2002). In short, 2 μg of total RNA from each sample was separated on denaturing formaldehyde agarose gels. The RNAs were transferred to Nylon membranes by downward capillary blotting and fixed by UV-crosslinking. Digoxigenin-labeled probes were generated by PCR using DIG-dUTP and a dNTP mix with a reduced dTTP concentration. The primers for probe generation are summarized in Supplementary Table S1. Hybridization was performed overnight at 50°C. The membranes were washed twice with 2 × SSC/0.1% SDS and twice in 1 × SSC/0.1% SDS. The probe was detected using an anti-DIG antibody coupled to alkaline phosphatase and the chemiluminescence substrate CDP-star according to the instruction of the manufacturer (Roche, Mannheim, Germany). The signals were visualized with X-ray films (GE Healthcare, Buckinghamshire, United Kingdom), and the sizes were analyzed with the marker “RiboRuler High Range RNA Ladder” (Thermo Fisher Scientific^1^).

### DNA Microarray Analysis

The transcriptomes of the wildtype H26 and the sRNA132 deletion mutants were compared using a home-built DNA microarray (Zaigler et al., 2003). RNA isolation, cDNA synthesis with RNase H minus M-MLV reverse transcriptase, labeling with Cy3-dUTP and Cy5-dUTP, respectively, hybridization, and washing have been described previously. Four biological replicates were performed, two of which with a reversal of the labeling dyes (day swap). The microarrays were scanned with the GenePix Pro 4200 (Molecular Devices, San Jose, CA, United States) and differential transcript levels were analyzed as described (Zaigler et al., 2003). For all spots the background signal was subtracted from the sample signal. All samples were removed with a signal of less than 100. The average intensities of the red and green signals in each of the four experiments were normalized to equity. The signal ratios were calculated for each spot. Only spots were included in the analysis that had signals in at least two of the four experiments. Averages of the signal ratios (or the reverse ratios for the dye swap experiments) and their standard deviations were calculated. All results are summarized in Supplementary Table S2. The experiment and the results have also been submitted to the ArrayExpress database^2^ and obtained the accession no. E-MTAB-7834.

### 5′-/3′-End Determination of Transcripts

The 5′-and 3′-ends of transcripts were determined as described previously (Brenneis et al., 2007). In short, total RNA was isolated and the 5′-and 3′-ends were ligated with T4 RNA ligase (New England Biolabs, Ipswich, MA, United States). After removal of the protein with phenol, a gene-specific primer was hybridized and cDNA was synthesized with M-MLV reverse transcriptase (RNase H minus; Promega, Fitchburg, WI, United States). The cDNA was amplified by PCR using two gene-specific primers, and two further primers were used for a nested PCR. All primer sequences are included in Supplementary Table S1. The sequence of the nested PCR product was then determined by GATC Biotech (Cologne, Germany). Comparison with the genome sequence enabled determination of the 5′-and the 3′-end of the transcripts.

### General Molecular Biological Methods

General molecular biology methods were performed using standard procedures (Green and Sambrook, 2012). Genomic DNA of *H. volcanii* was isolated according to Mevarech and Werczberger. For sequence analysis, 25 pMol of the primer and about 500 ng of the respective plasmid were mixed and sent to GATC Biotech (Cologne, Germany). *H. volcanii* was transformed as described previously (Cline et al., 1989).

### Databases and Computer Programs

Homologs of sRNA132 were identified in (draft) genomes of Halobacteriales (taxid: 2235) using nucleotide BLAST (Wolfsberg and Madden, 2001) at the NCBI website^3^. The following parameters were used: word size 28, expected threshold 10, match score 1, mismatch score -2, linear gap costs, automatically adjust parameters for short input sequences yes, highly similar sequences yes. Multiple sequence alignments were generated using LocaRNA, which allows the concomitant identification of conserved structures (Will et al., 2012). The following parameters were used: alignment type global, structure weight 200, indel opening score -500, indel score -350, match score 50, maximal base pair span 150, temperature 40°C, energy parameter set rna_turner2004.par, minimal pair probability 5.0E-4, minimal probability for constructing the guide tree 0.005, maximal differences for sizes of matched arcs 30, maximal differences for alignment edges 60. Putative interaction sites were predicted *in silico* using the IntaRNA webserver 2.3.0 (wrapper 1.1.5) given the following parameters: hybridization temperature: 42°C; folding window size: 150; maximum basepair distance: 100; minimum number of basepairs: 7; number of mismatch 1 suboptimal (Busch et al., 2008; Smith et al., 2010).

## Results

### Conservation and Importance of sRNA132

For reasons described above the sRNA132 was the first sRNA of *H. volcanii* that was chosen for a detailed characterization. Its 5′- and 3′-ends were determined by cRT-PCR and it was revealed that the sRNA132 has a length of 138 nt (genomic coordinates: 1 280 196–1 280 333). The same 5′-end was also found in a recent dRNA-Seq study (Babski et al., 2016), underscoring that it is a primary transcript and not processed from a larger precursor sRNA. In accordance with this view, the transcription start site (TSS) is preceded by the basal promoter elements BRE, TATA box, and -10 element (Supplementary Figure S1A). The downstream gene HVO_1405 is also preceded by basal promoter elements, and a transcription start site was found in the dRNA-Seq study (Babski et al., 2016), underscoring that it does not form an operon with the sRNA132 gene.

**FIGURE 1:**
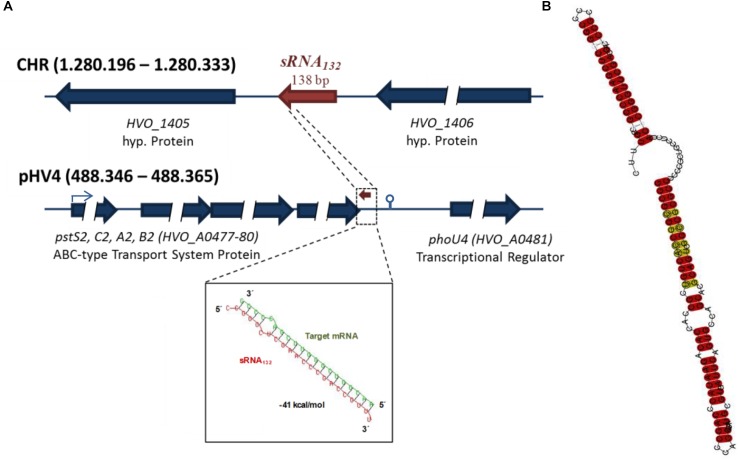
Features of sRNA132. **(A)** Genomic localizations of the sRNA132 gene and the operon of its putative target mRNA. HVO numbers are designations of genes in the *H. volcanii* genome. The predicted interaction between sRNA132 and the 3′-UTR of its target mRNA is shown at the bottom. **(B)** Predicted secondary structure of sRNA132. The prediction is based on a multiple sequence alignment (Supplementary Figure S1B). The degree of conservation is color-coded, highly conserved positions are shown in red.

A BLAST searched showed that homologous genes are present in more than ten species of *Haloferax*, but could not be found in other archaeal or bacterial genera. Supplementary Figure S1B shows a multiple sequence alignment of sRNA132 and its homologs and underscores the high degree of conservation. The predicted folding consists of two double-stranded stems, which are separated by a pyrimidine-rich single-stranded linker region of 13 nt (Figure 1B).

The sRNA132 is encoded on the major chromosome of *H. volcanii* between two genes for hypothetical proteins (Figure 1A). In 11 species (out of 12), its genomic localization between the two conserved hypothetical proteins is also conserved. This could either indicate a functional connection between the sRNA and these genes, or – more probably – that since its evolution within the genus *Haloferax* no genomic rearrangement have occurred between the sRNA gene and the adjacent genes.

The distances of 101 nt and 107 nt between the sRNA132 gene and these neighboring genes as well as the presence of basal promoter elements and transcriptional start sites (see above) show that it is transcribed as a monocistronic RNA. Transcriptional terminators are not well defined in Archaea, therefore, a search for the presence of a terminator at its 3′-end was not possible.

**FIGURE 2:**
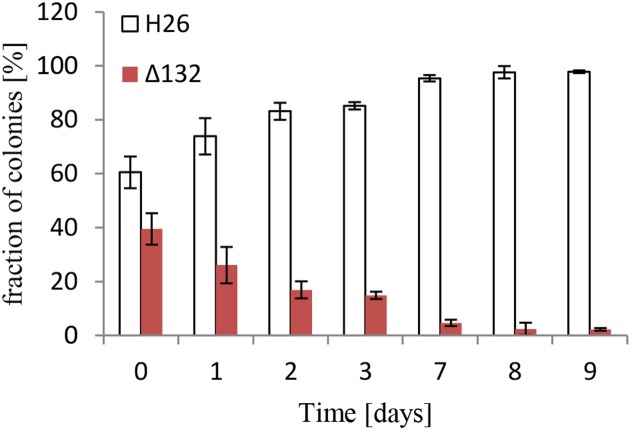
Competitive growth between the wild-type and a sRNA132 gene deletion mutant. The sRNA132 gene deletion mutant had the normal red color of *H. volcanii*. The sRNA132 wild-type was white due to the deletion of the carotenoid-biosynthesis gene HVO_2528. Both strains were grown to mid-exponential phase and mixed to nearly equal rations. The mixture was grown overnight, an aliquot was taken and dilutions were plated to enable quantification of the numbers of red and white colonies. The overnight culture was also used to inoculate a fresh culture, and this procedure was repeated for 9 days. The fractions of red and white colonies are shown.

In a previous study 28 sRNA gene deletion mutants had been constructed and phenotypically characterized at 10 different conditions, including sRNA132 (Jaschinski et al., 2014). Unfortunately, the analyses did not yield a clear indication of what the biological function of sRNA 132 might be. In this earlier study, simple growth curves had been used, which do not allow to identify small growth differences with high certainty. As an alternative approach, competitive growth experiments were performed. To this end, the carotenoid synthesis-proficient red sRNA132 deletion mutant was mixed with a white sRNA132 wild-type, which had a deletion in a carotenoid biosynthesis gene. The cell mixture was grown, and every day aliquots were removed to determine the fractions of red and white cells and to inoculate fresh cultures. The result is shown in Figure 2. The wild-type clearly outperformed the sRNA132 deletion mutant during competitive growth, showing that this sRNA must have an important biological function.

As a possible approach to identify genes that are influenced by sRNA 132, the transcriptomes of the sRNA132 deletion mutant and the wild-type were compared with a home-build DNA microarray using cultures grown under optimal conditions in synthetic medium with glucose to mid-exponential growth phase. Only very few genes exhibited differential transcript levels, including the HVO_A0477-A0480 operon, which is annotated to encode a phosphate-specific ABC transporter. A bioinformatics analysis revealed a very stable putative interaction between the sRNA132 and a sequence downstream of the stop codon of HVO_A0480, which could be part of the 3′-UTR of the operon transcript (Figure 1A). The transcript ends of the operon transcript were determined using cRT-PCR, and it was verified that the putative interaction site is indeed included in the 3′-UTR (compare the terminator motif in Figure 1A). Therefore, it was decided to unravel whether the sRNA132 has any effect on the expression of the operon HVO_A0477-A0480.

### Influence of sRNA132 on the Expression of an ABC Transporter Operon

**FIGURE 3:**
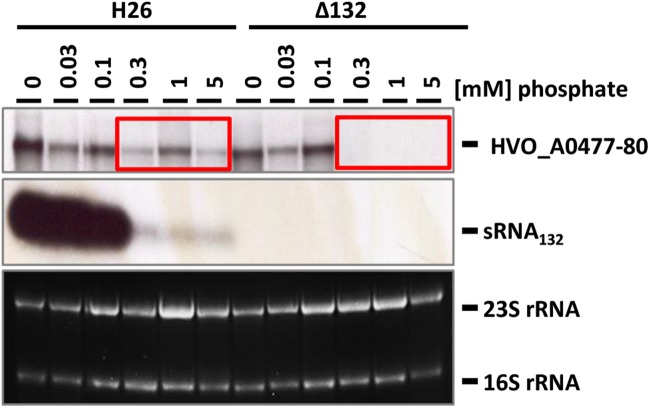
Transcript levels of sRNA132 and the HVO_A0477-A0480 operon mRNA at different phosphate concentrations. The operon encodes a phosphate-specific ABC transporter. The wild-type H26 and the sRNA132 gene deletion mutant were grown in media with various phosphate concentrations, as indicated at the top. At mid exponential growth phase (about 5 × 10^8^ cells ml^-1^) aliquots were removed, RNA was isolated and used for Northern blot analyses with operon-specific and sRNA-specific probes, as indicated at the right. Ethidium bromide stained 23S and 16S rRNAs are shown at the bottom. The two red boxes highlight phosphate concentrations at which the operon mRNA was found only in the presence of the sRNA132.

The wild-type and the sRNA132 deletion strain were grown overnight in synthetic medium with various phosphate concentrations from 0 to 5 mM, and the levels of the sRNA and its putative target transcript HVO_A0477-A0480 were determined with Northern blot analyses (Figure 3). It was verified that the deletion mutant was devoid of the sRNA. In the wild-type the sRNA was highly induced at very low phosphate concentrations from 0 to 0.1 mM, while the level was much lower at phosphate concentrations of 0.3–5 mM. At the latter phosphate concentrations the operon transcript was only detectable in the wild-type and was missing from the sRNA deletion mutant, indicating that the sRNA has a positive regulatory effect on the mRNA (compare boxes in Figure 3). Unexpectedly, the amounts of the operon transcript were the same in the deletion mutant and the wild-type at very low phosphate concentrations. This indicated that the expression of the HVO_A0477-A0480 operon is regulated by at least two different mechanisms, and that only one of them is sRNA132-dependent. In contrast, the other mechanism does not require the sRNA, and it is tempting to speculate that it could be differential transcriptional regulation (see section “Discussion”).

**FIGURE 4:**
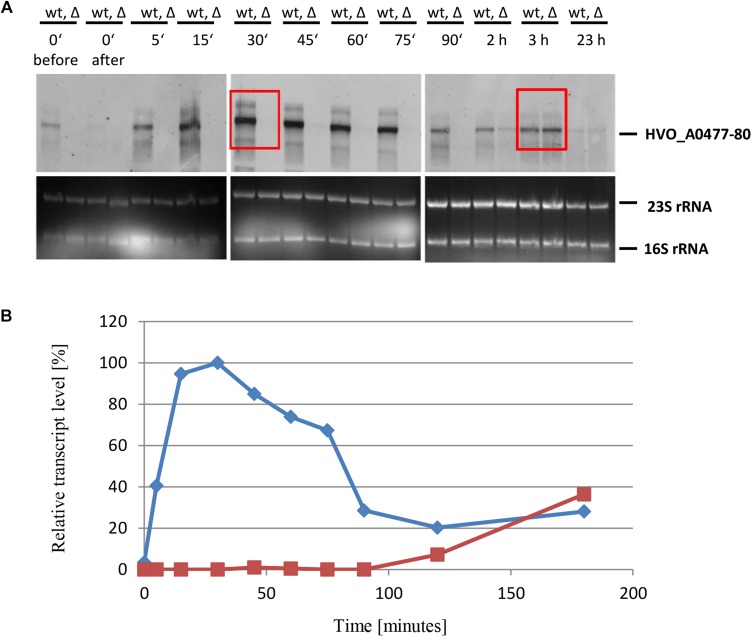
Kinetic analysis of the HVO_ A0477-A0480 operon mRNA level after the onset of phosphate starvation. **(A)** The operon encodes a phosphate-specific ABC transporter. The wild-type (wt) H26 and the sRNA132 gene deletion mutant (Δ) were grown to mid-exponential growth phase in the presence of phosphate (1 mM). The cells were harvested and resuspended in phosphate-free medium. At the time points indicated at the top aliquots were removed, RNA was isolated and used for Northern blot analysis with an operon-specific probe. Ethidium bromide stained 23S and 16S rRNAs are shown at the bottom. The two red boxes highlight time points discussed in the text. **(B)** The signals of the Northern blots were quantified densitometrically, and the kinetic changes of relative transcript levels are shown.

To characterize the role of sRNA132 in phosphate-dependent regulation further, the wild-type and the sRNA deletion mutant were grown in the presence of phosphate sufficiency (1 mM phosphate) and were then shifted to phosphate starvation conditions. At different time points aliquots were removed and the level of the HVO_A0477-A0480 transcript was determined by Northern blot analysis (Figure 4A). A densitometric analysis war performed to quantify the transcript level changes (Figure 4B). It was revealed, that shortly after the onset of phosphate starvation, the presence of the sRNA132 was essential for the operon transcript. In the absence of the sRNA the operon transcript was undetectable for 90 min, and only after 120 min a very faint signal could be detected, showing that the sRNA-independent regulatory mechanism of induction is very slow. In stark contrast, in the presence of the sRNA (wild-type) the operon transcript was already present 5 min after the onset of phosphate starvation, and its level peaked at around 30 min (left box in Figure 4A). After 180 min, the levels of the operon transcript were indistinguishable in the wild-type and the sRNA deletion mutant (right box in Figure 4A). Taken together, the sRNA132 mediated a very fast and transient induction of the HVO_A0477-A0480 transcript, and thus it seemed to be indispensable for the adaptation to rapidly changing environments.

### Elucidation of the sRNA132 Regulon After the Onset of Phosphate Starvation

The importance of the sRNA132 for the expression of the operon was highest about 30 min after the onset of phosphate starvation, therefore, the transcriptomes of the wild-type and the sRNA deletion mutant were compared at this time point using the home-build DNA microarray. The DNA microarray was originally generated from PCR fragments of 1.5 kbp, and thus each spot represents, on average, about 1.5 genes (Zaigler et al., 2003). Subsequently, oligonucleotides have been added that address a single coding or non-coding transcript. Four biological replicates were performed, including two experiments with a dye swap. All results are summarized in Supplementary Table S2, and they have been submitted to the ArrayExpress database and got the accession No. E-MTAB-7834. In total, 1721 spots could be analyzed in at least one experiment, 1336 spots in at least two experiments, 1014 in at least three experiments, and 691 in all four experiments. Spots that gave a signal in only one experiment were excluded from the analysis, and, thus, the analysis was restricted to 1336 spots. Transcripts were regarded as differentially regulated between the sRNA132 deletion mutant and the wildtype when the average signal ratio was lower than 0.5 or higher than 2.0. In total 23 spots represented downregulated genes (Supplementary Table S3). Six spots with the lowest average ratios represented the HVO_A0477-A0480 operon, underscoring the importance of sRNA132 for its expression shortly after the onset of phosphate starvation. In addition, a second ABC transporter operon, HVO_2375-2378, was also down-regulated in the absence of the sRNA. The list contains various further genes encoding proteins involved in phosphate metabolism. Examples are an ABC transporter for glycerol-3-phosphate, a polyphosphate kinase, and a glycerolphosphate-diesterase, which all have lower transcript levels in the sRNA deletion mutant (Supplementary Table S3).

However, 31 spots were also regulated in the opposite direction and had more than twofold higher transcript levels in the sRNA deletion mutant, indicating that sRNA132 is a negative regulator of these genes (Supplementary Table S4). These include glycerol-1-phosphate dehydrogenase and glycerol-3-phosphate dehydrogenase, two additional proteins involved in phosphate metabolism. Remarkably, the list of genes that were up-regulated in the sRNA deletion mutant contained four CPXCG zinc finger μ-proteins and thus this gene family is enriched among the sRNA-regulated genes.

The 51 up- or downregulated spots represent 3.8% of the 1336 analyzed spots of the DNA microarray, indicating that the vast majority of 96% of genes are not differentially regulated between the sRNA132 deletion mutant and wildtype (Supplementary Figure S2). Because several spots represent the same genes, the 51 spots represent about 40 genes and operons. It should also be noted that many biological functions are missing from the sRNA132-regulated genes, e.g., ribosomal proteins and other translational proteins and proteins of the basal transcription machinery. These results underscored that sRNA132 influences the expression of a dedicated regulon with relevance to phosphate metabolism. To deepen the insight, the sRNA132-dependent differential expression was analyzed for the second phosphate-specific ABC transporter and four further members of the regulon.

### Influence of sRNA132 on the Expression of a Second ABC Transporter Operon

**FIGURE 5:**
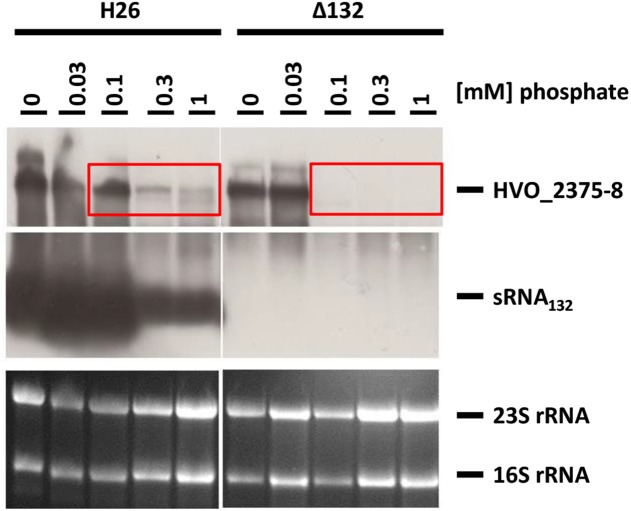
Transcript levels of sRNA132 and the HVO_2375-2378 operon mRNA at different phosphate concentrations. The operon encodes a phosphate-specific ABC transporter. The wild-type H26 and the sRNA132 gene deletion mutant were grown in media with various phosphate concentrations, as indicated at the top. At mid exponential growth phase (about 5 × 10^8^ cells ml^-1^) aliquots were removed, RNA was isolated and used for Northern blot analyses with operon-specific and sRNA-specific probes, as indicated at the right. Ethidium bromide stained 23S and 16S rRNAs are shown at the bottom. The red boxes highlight phosphate concentrations at which the operon mRNA could only be detected in the presence of sRNA132.

The dRNA-Seq results (Babski et al., 2016) and RNA-Seq results (Laass et al., 2019) revealed the existence of a polycistronic transcript of the operon HVO_2375-2378, which is leaderless and has a typical 3′-UTR of about 50 nt. The wild-type and the sRNA132 deletion mutant were grown in synthetic medium with different phosphate concentrations, and the levels of the sRNA and the HVO_2375-2378 operon transcript were determined by Northern blot analyses (Figure 5). The results for this second phosphate-specific ABC transporter were very similar to the results for the first ABC transporter described above. Again, the sRNA level was highly induced at very low phosphate concentrations (0.1 mM and lower). Again, under steady state growth conditions, the operon transcript was present at very low phosphate concentrations, irrespective of the presence of the sRNA. And again, at slightly higher phosphate concentrations (0.1–1.0 mM) the operon transcript was present in the wild-type, but was undetectable in the sRNA mutant, verifying the DNA microarray results that sRNA132 is a positive regulator of the HVO_2375-2378 operon.

**FIGURE 6:**
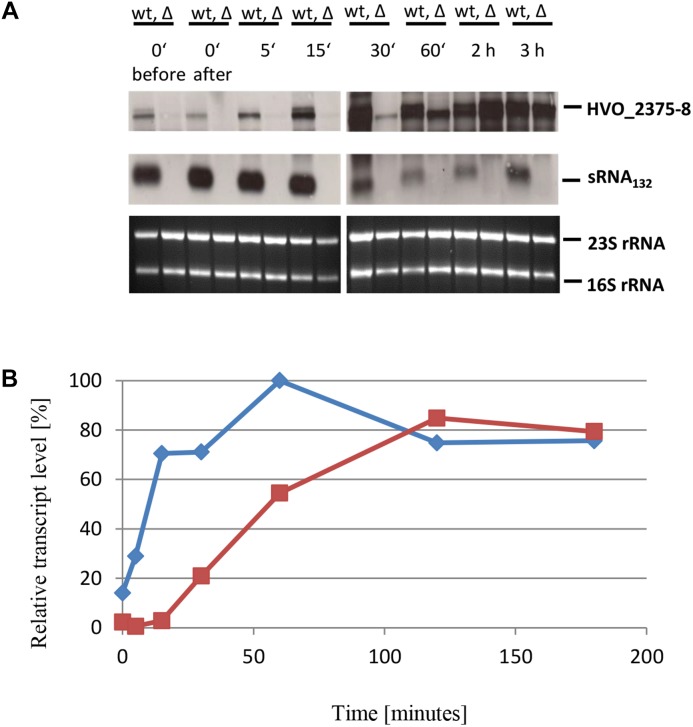
Kinetic analysis of the HVO_ 2375-2378 operon mRNA level after the onset of phosphate starvation. **(A)** The operon encodes a phosphate-specific ABC transporter. The wild-type (wt) H26 and the sRNA132 gene deletion mutant (Δ) were grown to mid-exponential growth phase in the presence of phosphate (1 mM). The cells were harvested and resuspended in phosphate-free medium. At the time points indicated at the top aliquots were removed, RNA was isolated and used for Northern blot analysis with an operon-specific and a sRNA-specific probe. Ethidium bromide stained 23S and 16S rRNAs are shown at the bottom. **(B)** The signals of the Northern blots were quantified densitometrically, and the kinetic changes of relative transcript levels are shown.

The wild-type and the sRNA132 deletion mutant were also grown to mid-exponential phase in the presence of 1 mM phosphate and then shifted to phosphate starvation conditions. Northern analyses were performed (Figure 6A) and the transcript level changes were quantified densitometrically (Figure 6B). It was revealed that shortly after the onset of starvation the presence of the operon transcript strictly depended on the presence of the sRNA132, and it was totally lacking in the sRNA deletion mutant. However, the sRNA-independent induction of the expression of this operon began slightly earlier than that of the first operon described above, a faint signal of the HVO_2375-2378 transcript was already visible 30 min after the start of starvation. At later time points (2 and 3 h) the level of the operon transcript was the same in the presence and the absence of the sRNA. This kinetic analysis identified HVO_2375-2378 as a second operon that needed the sRNA132 for a fast and transient induction of expression, before a second regulatory mechanism took over.

### Importance of the Two ABC Transporters for the Growth at Low P Concentrations

**FIGURE 7:**
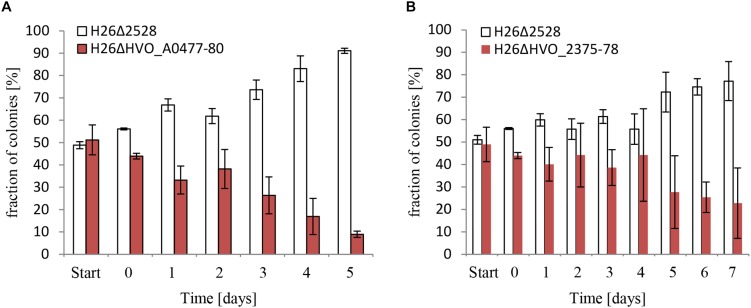
Competitive growth between the wild-type, and respectively, a HVO_A0477-A0480 operon deletion mutant **(A)** and a HVO_2375-2378 operon deletion mutant **(B)**. The operon deletion mutants had the normal red color of *H. volcanii*. The operon wild-types were white due to the deletion of the carotenoid-biosynthesis gene HVO_2528. For comparative growth analysis two strains were grown to mid-exponential phase and mixed to equal rations. The mixture was grown overnight, with very reduced phosphate concentrations of 10 μM **(A)** and 50 μM **(B)**, instead of the normal concentration of 1 mM. Aliquots were taken and dilutions were plated to enable quantification of the numbers of red and white colonies. The overnight culture was also used to inoculate a fresh culture, and this procedure was repeated for 5 and 7 days, respectively. The fractions of red and white colonies are shown.

Next, the biological importance of the two putative phosphate-specific ABC transporters was addressed. To this end, deletion mutants of the two operons were generated in a background that was proficient for carotenoid biosynthesis and thus formed red cells. Cultures of the two mutants and the carotenoid synthesis-deficient white wild-type were grown to mid-exponential phase in the presence of 1 mM phosphate. Then equal mixtures of the wild-type and each of the mutants were generated and were grown competitively at a very low phosphate concentration of 50 μM. Every day aliquots of the mixtures were transferred to fresh medium with 50 μM phosphate to continue competitive growth. In addition, every day aliquots were diluted and plated to enable the quantification of red and white colonies. The result is shown in Figure 7A for HVO_A0477-A0480 and in Figure 7B for HVO_2375-2378. In both cases, the wild-type very rapidly outgrew the respective operon deletion mutant, showing the high importance of the two phosphate-specific ABC transporters.

A double deletion mutant of both ABC transporter operons could be generated in complex medium. However, the mutant did not grow at all in synthetic medium, neither with the normal phosphate concentration of 1 mM, nor with elevated phosphate concentrations of 5 or 10 mM. Therefore, the presence of at least one of the two phosphate-specific ABC transporter operons is essential in synthetic medium. This is different in complex medium, which contains organic phosphate sources.

### Differential Expression of Additional Members of the sRNA132 Regulon

**FIGURE 8:**
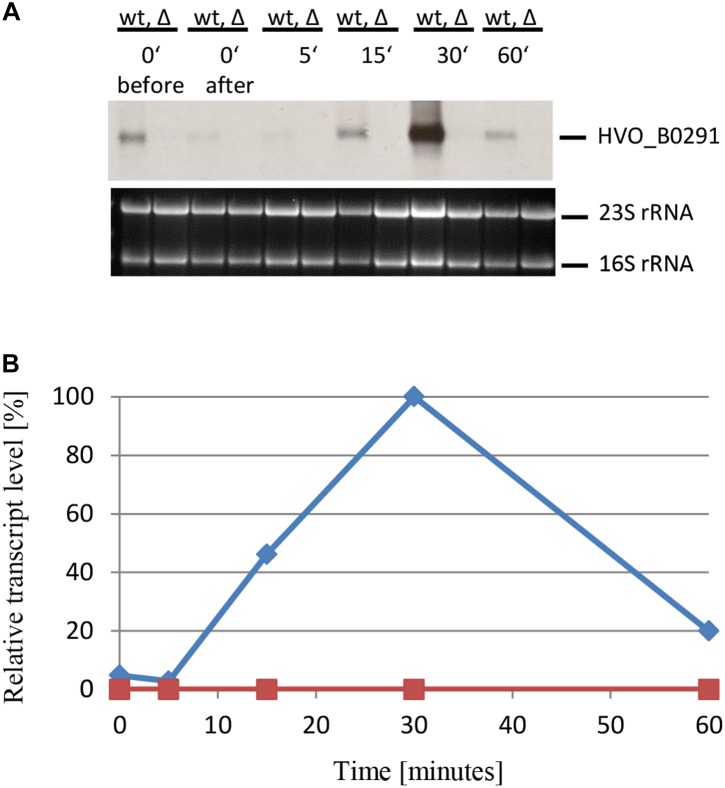
Kinetic analysis of the HVO_B0291 mRNA level after the onset of phosphate starvation. **(A)** HVO_B0291 encodes a glycerophosphate diesterase. The wild-type (wt) H26 and the sRNA132 gene deletion mutant (Δ) were grown to mid-exponential growth phase in the presence of phosphate (1 mM). The cells were harvested and resuspended in phosphate-free medium. At the time points indicated at the top aliquots were removed, RNA was isolated and used for Northern blot analysis with an HVO_B0291-specific probe. Ethidium bromide stained 23S and 16S rRNAs are shown at the bottom. **(B)** The signals of the Northern blots were quantified densitometrically, and the kinetic changes of relative transcript levels are shown.

Four additional genes were chosen to verify that sRNA132 regulates further genes detected by the DNA microarray analysis and to analyze whether the importance of sRNA132 is transient also for other members of the regulon. First, HVO_B0292 was chosen, which is part of an operon encoding an ABC transporter with glycerol-3-phosphate as annotated substrate (HVO_B0292-B0295). Northern blot analysis revealed that sRNA132 had a positive effect also on the level of this transcript, the effect was also transient, and the largest effect was observed after 30 min phosphate starvation (data not shown). Next, the gene directly upstream of the ABC transporter operon, HVO_B0291, was chosen. It encodes a glycerophosphate diesterase, which could liberate orthophosphate from the imported glycerophosphate. Again, the presence of RNA132 in the wildtype had a large, transient, positive effect on the mRNA level (Figure 8A,B). In this case, the mRNA level was much lower at the time points 15 and 60 min, and, thus, the peak of the sRNA132 effect at 30 min was much sharper for HVO_B0291 than for the first three examples. In the sRNA deletion mutant the mRNA could not be detected at all, indicating the high importance of sRNA132 for the upregulation of the glycerolphosphate esterase shortly after the onset of phosphate starvation.

**FIGURE 9:**
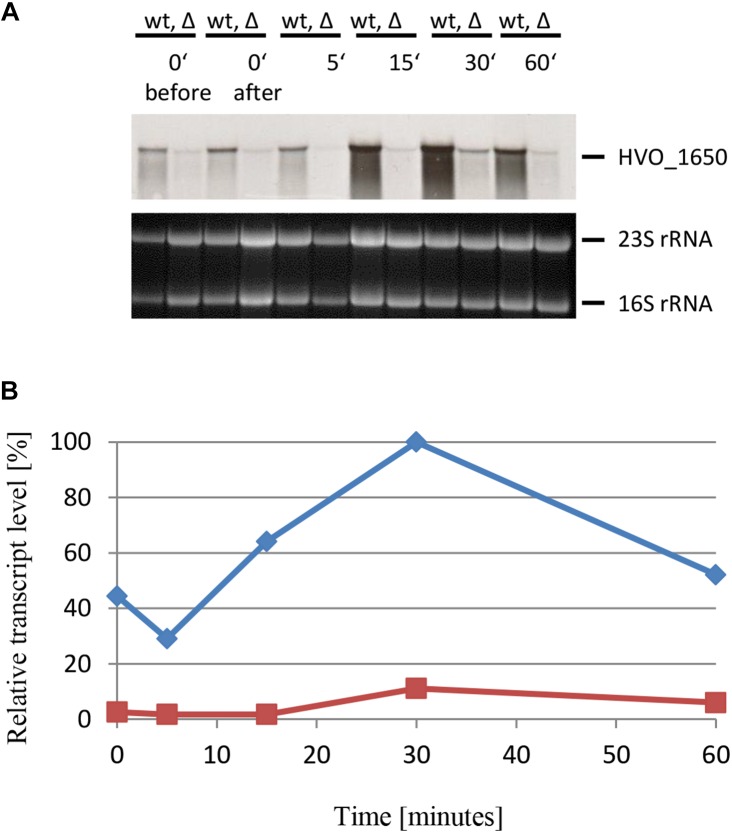
Kinetic analysis of the HVO_1650 mRNA level after the onset of phosphate starvation. **(A)** HVO_1650 encodes a polyphosphate kinase. The wild-type (wt) H26 and the sRNA132 gene deletion mutant (Δ) were grown to mid-exponential growth phase in the presence of phosphate (1 mM). The cells were harvested and resuspended in phosphate-free medium. At the time points indicated at the top aliquots were removed, RNA was isolated and used for Northern blot analysis with an HVO_1650-specific probe. Ethidium bromide stained 23S and 16S rRNAs are shown at the bottom. **(B)** The signals of the Northern blots were quantified densitometrically, and the kinetic changes of relative transcript levels are shown.

The next example was HVO_1650, which encodes a polyphosphate kinase. The experimental design was the same as before, and the results are shown in Figure 9A,B. As expected, based on the DNA microarray results, sRNA132 had a positive regulatory effect also on the HVO_1650 mRNA. The peak was again at 30 min after the onset of phosphate starvation, but the distribution was broader than seen for the last example, i.e., high levels were also seen at time points 15 and 60 min.

**FIGURE 10:**
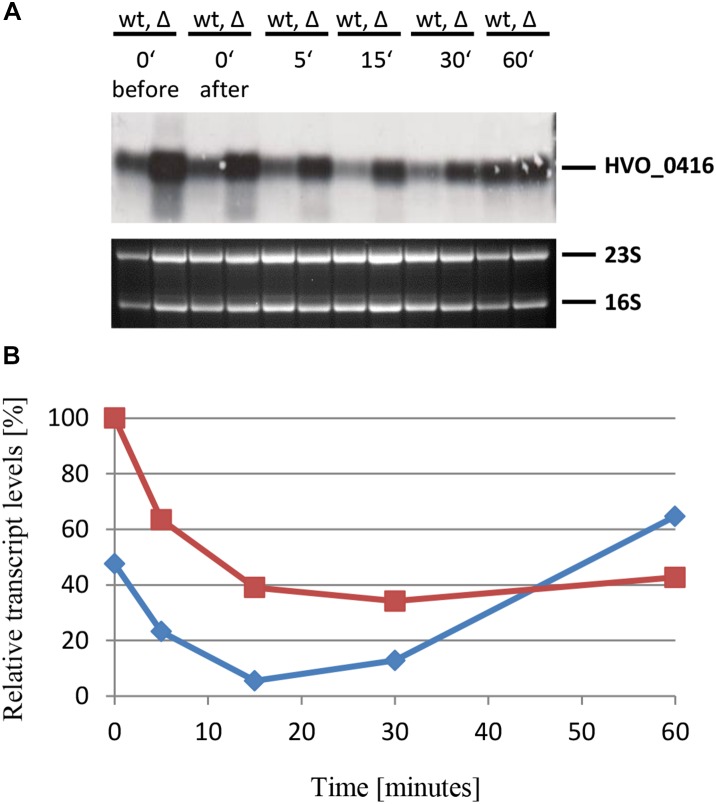
Kinetic analysis of the HVO_0416 mRNA level after the onset of phosphate starvation. **(A)** HVO_0416 encodes a small CPXCG zinc finger protein. The wild-type (wt) H26 and the sRNA132 gene deletion mutant (Δ) were grown to mid-exponential growth phase in the presence of phosphate (1 mM). The cells were harvested and resuspended in phosphate-free medium. At the time points indicated at the top aliquots were removed, RNA was isolated and used for Northern blot analysis with an HVO_0416-specific probe. Ethidium bromide stained 23S and 16S rRNAs are shown at the bottom. **(B)** The signals of the Northern blots were quantified densitometrically, and the kinetic changes of relative transcript levels are shown.

The last example that was studied by Northern blot analysis was the gene HVO_0416. It was chosen because the DNA microarray results had shown that it is negatively regulated by sRNA132, in contrast to the five examples described above. It encodes one of the four CPXCG zinc finger μ-proteins that are negatively regulated by sRNA132. The same experimental design was used, and the results are shown in Figure 10A,B. As expected, the mRNA level was indeed lower in the presence of the sRNA (wild-type) than in its absence. Also in this case the effect was transient, and the largest difference between the wild-type and the sRNA deletion mutant was seen at the time points 15 min and 30 min. Already after 60 min the HVO_0416 mRNA levels were identical, irrespective of the presence or absence of the sRNA, showing that another, slower regulatory mechanism takes over. In contrast to the other examples, there is no time point at which the mRNA was absent, and thus the sRNA132 modulated the mRNA level, but did not diminished it totally.

Taken together, Northern blot analyses were used to verify the regulatory role of sRNA132 on six of the about 40 differentially regulated transcripts detected by DNA microarray analysis. In all six cases the effect of sRNA132 was transient and peaked around 30 min after the onset of phosphate starvation. In addition, the direction of regulation was verified and it was shown that sRNA132 can have a positive as well as a negative regulatory effect on various mRNAs.

## Discussion

### sRNA Targets in Archaea

In recent years RNA-Seq analyses and dRNA-Seq analyses have led to the detection of a large number of non-coding RNAs in various species of Archaea, e.g., *H. volcanii* (Babski et al., 2016; Gelsinger and DiRuggiero, 2018b; Laass et al., 2019), *M. mazei* (Jäger et al., 2009), *P.* *abyssi* (Toffano-Nioche et al., 2013), *S. solfataricus* (Wurtzel et al., 2010), and *T. kodakarensis* (Jäger et al., 2014). However, until now only very few studies exist that unraveled the biological roles and/or identified the target mRNAs. Because bacterial sRNAs often bind to the 5′-regions of their target mRNAs, while, in contrast, eukaryotic miRNAs typically bind to the 3′-UTRs of their target mRNAs, it seems especially interesting to clarify the mode of action of several archaeal sRNAs.

The first target identification was performed with sRNA162 from *M. mazei* Gö1 (Jäger et al., 2012). It was shown that sRNA162 regulates two mRNAs, it acts in *cis* on mRNA MM2442 as well as in *trans* on mRNA MM2441. In both cases sRNA162 occludes the ribosome binding site (RBS) and the start codon, and, thereby, represses translation of the two mRNAs. Overexpression of sRNA162 led to transcript level changes of 185 genes (Jäger et al., 2012). Recently two additional sRNAs of *M. mazei* Gö1 have been characterized. Overexpression of sRNA154 resulted in transcript level changes of about 60 genes (Prasse et al., 2017). Interestingly, it was found that sRNA154 had two different mechanisms of action. On the one hand it enhanced the stability of several transcripts, including the *nifH* transcript, which encodes nitrogenase, an essential enzyme for nitrogen fixation. On the other hand, it occluded the RBS of the *glnA1* mRNA, resulting in an inhibition of translation (Prasse et al., 2017). A third sRNA of *M. mazei*, sRNA41, was also shown to occlude the RBS of several genes and downregulate translation initiation (Buddeweg et al., 2018). The deletion of the sRNA41 gene resulted in elevated levels of 36 proteins. Therefore, the results from the first three analyzed examples indicate that sRNAs in methanogenic archaea (1) typically downregulate translation by occluding the RBS and the start codon, and (2) directly or indirectly influence the levels of many transcripts and/or proteins.

In contrast, characterization of sRNA257 from *S. solfataricus* revealed that it acts by binding to the 3′-UTR of its target gene Sso1183, which encodes a putative phosphate transporter (Märtens et al., 2013). An inverse correlation between the levels of the sRNA and the mRNA indicated that sRNA257 is a negative regulator of the stability of mRNA Sso1183. In agreement with the predicted function of Sso1183, phosphate limitation resulted in a reduced level of sRNA257 and an enhanced level of mRNA Sso1183.

### Putative Targets of sRNA132

Characterization of sRNA132 of *H. volcanii* revealed some similarities, but also considerable differences to the four previously characterized sRNAs from *M. mazei* Gö1 and *S. solfataricus*. The DNA microarray analysis after 30 min of phosphate starvation revealed that sRNA132 influences the transcript levels of a large regulon of about 40 genes. Because the effects can be exerted either by direct binding to target mRNAs, or they can be indirect via other sRNAs or via regulatory proteins, putative interaction sites between sRNA132 and the possible target mRNAs have been predicted using IntaRNA (Supplementary Table S5). The results revealed that 25 putative interactions have energies of lower than -10 kJ/mol, and thus all these mRNAs are probably direct targets of sRNA132. Most of the remaining predicted interactions have binding energies between -7 and -10 kJ/mol, and thus many of these mRNAs might also be direct targets. In most cases the predicted interaction site of the sRNA132 either includes either the region 30–45, or the region 80–95, indicating that the sRNA has two different interaction regions, like sRNA154 from *M. mazei* Gö1. However, in contrast to *M. mazei* Gö1, the predicted interaction sites on the target mRNAs are not at the 5′-ends, but within the ORFs or within the 3′-UTR. This difference is in agreement with the fact that mRNAs in methanogenic archaea typically have extended 5′-UTRs and include Shine Dalgarno motifs (Jäger et al., 2009), while 72% of transcripts of *H. volcanii* are leaderless and thus lack SD motifs (Babski et al., 2016). Future experimental approaches are needed to validate the predicted interaction partners and interaction regions of sRNA132.

### Comparison of the sRNA132 Regulon of *H. volcanii* With the Phosphate Stimulon of *H. salinarum*

**FIGURE 11:**
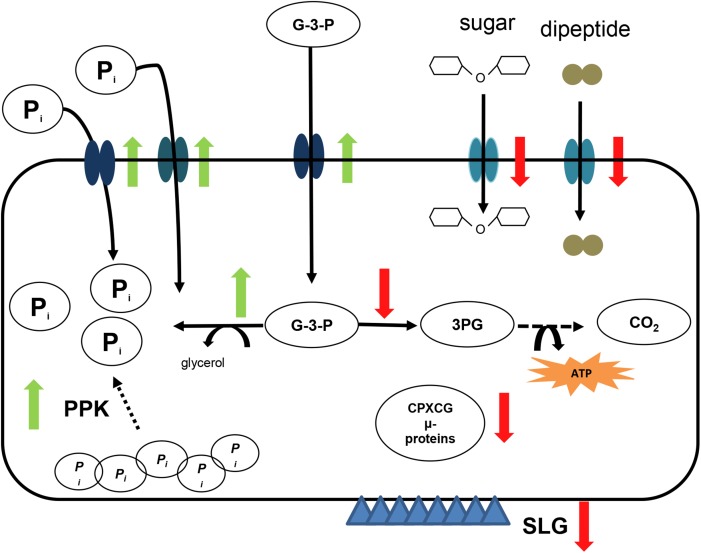
Schematic overview of the sRNA132 regulon. Selected members of the sRNA132 regulon are shown schematically and the positive or negative regulation by sRNA132 is indicated by green upward and red downward arrows, respectively. The following abbreviations are used: P_i_ – orthophosphate, G-3-P – glycerol-3-phosphate, 3PG – 3-phosphoglycerate, SLG – surface layer glycoprotein, PPK – polyphosphate kinase. CPXCG denotes an amino acid motif of zinc finger protein. The S-layer is indicated by blue triangles.

In any case, sRNA132 regulates many genes, a subset of which is related to phosphate metabolism, and specifically to the adaptation to phosphate starvation. Figure 11 gives an overview of selected functions, which are positively or negatively regulated by sRNA132 after the onset of phosphate starvation. Importantly, positively regulated gene products included three different ABC transporters, which can accumulate inorganic phosphate and glycerol-3-phosphate, and a glycerophosphate diesterase, which can liberate phosphate in the cytoplasm. Another gene encodes a putative polyphosphate kinase. In principle, the enzyme could increase the phosphate concentration if the cells would contain high concentrations of polyphosphate. However, *H. volcanii* does not contain polyphosphate under the applied growth conditions (Zerulla et al., 2014).

The DNA microarray analysis also identified genes that were downregulated by sRNA132. Selected downregulated genes encode two ABC transporters with peptides and sugars as predicted substrates, a dehydrogenase, which funnels imported glycerol-3-phosphate in the direction of energy metabolism, and the surface layer glycoprotein, which forms the S-layer of the cell. This regulatory pattern is in agreement with the observed growth retardation after the onset of phosphate starvation.

Comparison of the transcriptomes of cultures grown in the presence versus the absence of phosphate was used to unravel the phosphate stimulon of the haloarchaeon *H. salinarum* (Wende et al., 2009). The upregulated genes under phosphate starvation encode two ABC transporters for inorganic phosphate, an ABC transporter for glycerol-3-phosphate, a glycerolphosphate diesterase, and only a few other genes (Table 2 and Supplementary Table S4 in Wende et al., 2009). Therefore, there is a large overlap between the upregulated genes in the sRNA132 regulon of *H. volcanii* and the phosphate stimulon of *H. salinarum*. However, the induction of all those genes is much slower in *H. salinarum*, none of the transcript levels is significantly induced even 2 h after the onset of phosphate starvation, in stark contrast to the pronounced transcript level changes in *H. volcanii* after only 15 min (Figure 4, 6, 8–10). Notably, *H. salinarum* does not contain a homolog of sRNA132. It has been argued that a major advantage of sRNA-based regulation, compared to the regulation by transcription factors, is a much faster response (Wagner and Romby, 2015; Holmqvist and Wagner, 2017). The fast regulation by sRNA132 seems to be a perfect example to underscore this theory. While sRNA132 is exclusively present in the genus *Haloferax*, the transcriptional regulation by PhoU transcription factors is conserved in haloarchaea. Haloarchaea typically contain at least one gene encoding a PhoU regulator, and *H. salinarum* and *H. volcanii* contain four *phoU* genes. For *H. salinarum* it has been experimentally shown that pho-boxes, the binding motifs of PhoU regulators, are present in the promoters of the two phosphate specific ABC transporters, and that they are essential for transcriptional upregulation (Furtwängler et al., 2010). The restricted presence of sRNA132 indicates that is has been newly evolved in the genus *Haloferax*, and this exemplifies another advantage of sRNA-based regulation, namely, its faster evolution compared to protein-based regulation.

### Transient Regulation of Gene Expression in Archaea

A unique feature of sRNA132 is its transient importance within the first hour after the onset of phosphate starvation, before another regulatory mechanism takes over. To our knowledge, this has never been described before for any archaeal or bacterial sRNA. However, transient effects have been reported previously for protein-coding genes. For example, more than 30 genes were transiently induced in *H. volcanii* after a shift from casamino acids to glucose, but had the same expression level under steady state conditions in both carbon sources (Zaigler et al., 2003). Transient transcript level regulation was also observed in *Pyrococcus furiosus* as a response to a temperature down-shift (Weinberg et al., 2005). Therefore, it seems that transient regulation of gene expression after a change of conditions might be more wide-spread than anticipated. It has even been described that transcript levels oscillate in *H. salinarum* under constant conditions, after an entrainment period of light dark cycles (Whitehead et al., 2009). And, of course, cell cycle-specific transcript levels changes represent well-known transient changes under constant external conditions, which have been analyzed for *H. salinarum* (Baumann et al., 2007) and *Sulfolobus acidocaldarius* (Lundgren and Bernander, 2007).

On more than half of its putative targets sRNA132 acts as a positive regulator. Positive regulation has also been described for various bacterial sRNAs (Papenfort and Vanderpool, 2015). One mechanism is the occlusion of a recognition site for a single-strand specific RNase by the sRNA. In archaea, stabilization of an RNA via duplex formation has recently been described for *S. acidocaldarius* (Orell et al., 2018). Seven genes in the genome of *H. volcanii* are annotated to encode RNases of different types, which might be involved in regulation. Future studies are required to unravel the molecular details of the regulatory mechanism of sRNA132, including the clarification whether any of the seven annotated RNases is involved.

## Conclusion

An in depth analysis of the function of sRNA132 of *H. volcanii* revealed that it regulates an extended regulon of about 40 genes. Bioinformatics target site predictions indicate that sRNA132 can interact directly with the majority of regulon members. Expression of sRNA132 is highly induced during phosphate limitation, and many of the regulon members are involved in phosphate metabolism. sRNA132 is the first example of an archaeal or bacterial regulatory sRNA that acts transiently after the onset of starvation, while another mechanism, most probably regulation of transcription, subsequently takes over.

## Author Contributions

JK and JS conceived the study. JK and KJ performed the experiments. JK, KJ, and JS interpreted the data. JK and JS wrote the manuscript.

## Conflict of Interest Statement

The authors declare that the research was conducted in the absence of any commercial or financial relationships that could be construed as a potential conflict of interest.

## Footnotes

1 http://www.thermofisher.com

2 https://www.ebi.ac.uk/arrayexpress/

3 https://blast.ncbi.nlm.nih.gov
