# Supplementary figures and images for: A Haloarchaeal Small Regulatory RNA (sRNA) Is Essential for Rapid Adaptation to Phosphate Starvation Conditions
Source: Front Microbiol. 2019 Jun 5;10:1219. doi: 10.3389/fmicb.2019.01219 (PMC6560208; doi:10.3389/fmicb.2019.01219)

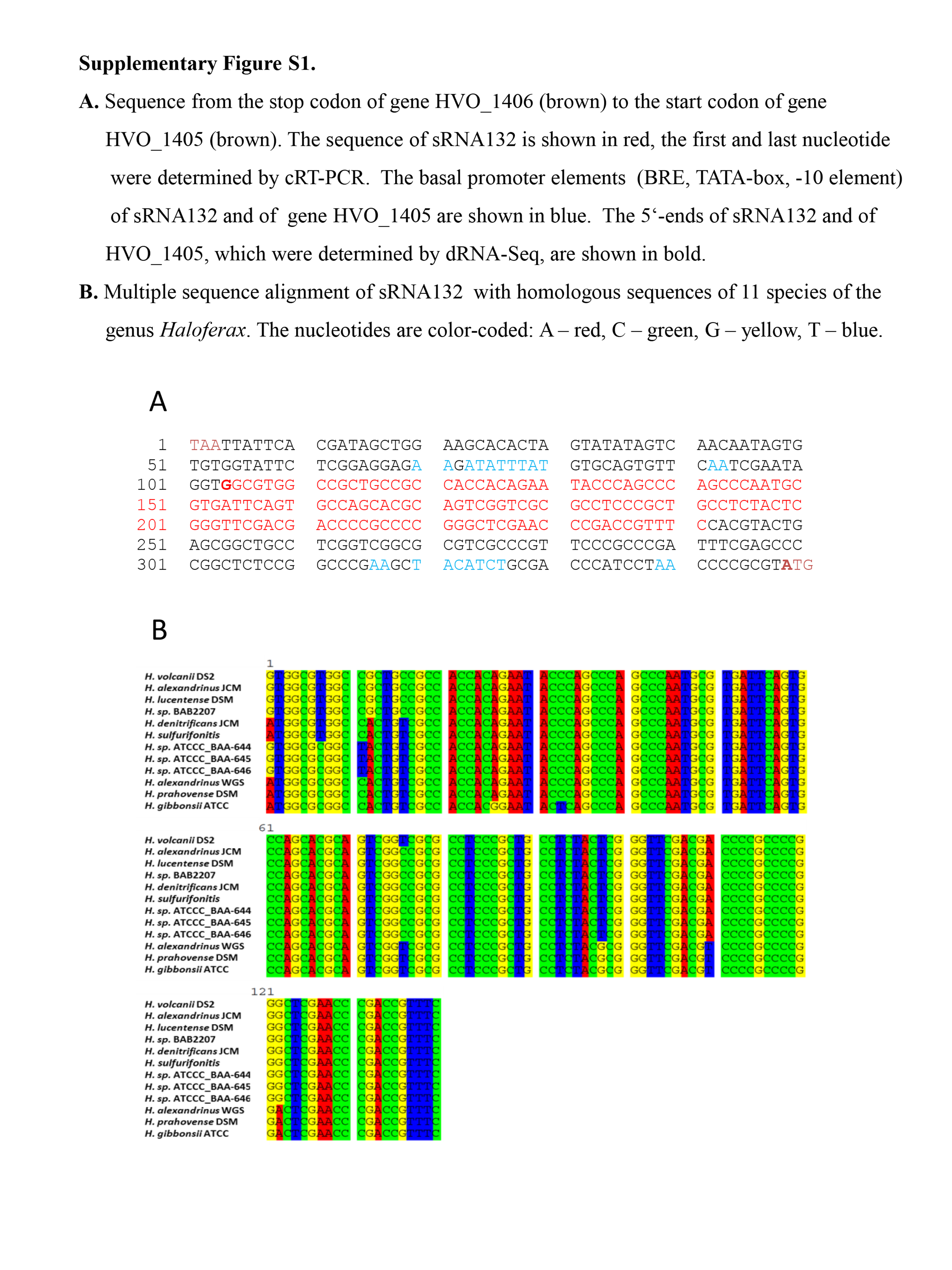

Supplement: Supplementary file 6 [file Image_1.TIF]

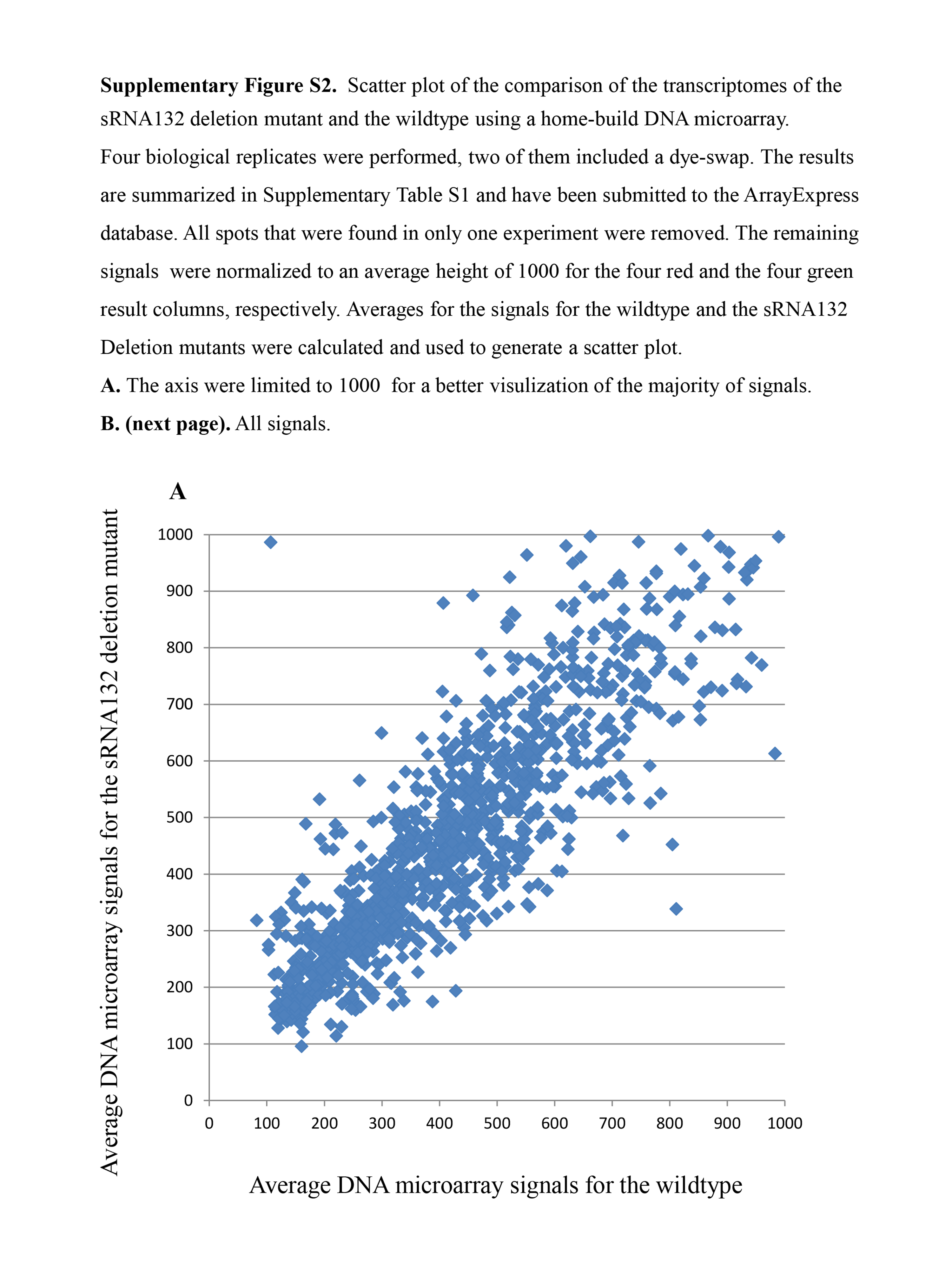

Supplement: Supplementary file 7 [file Image_2.TIF]

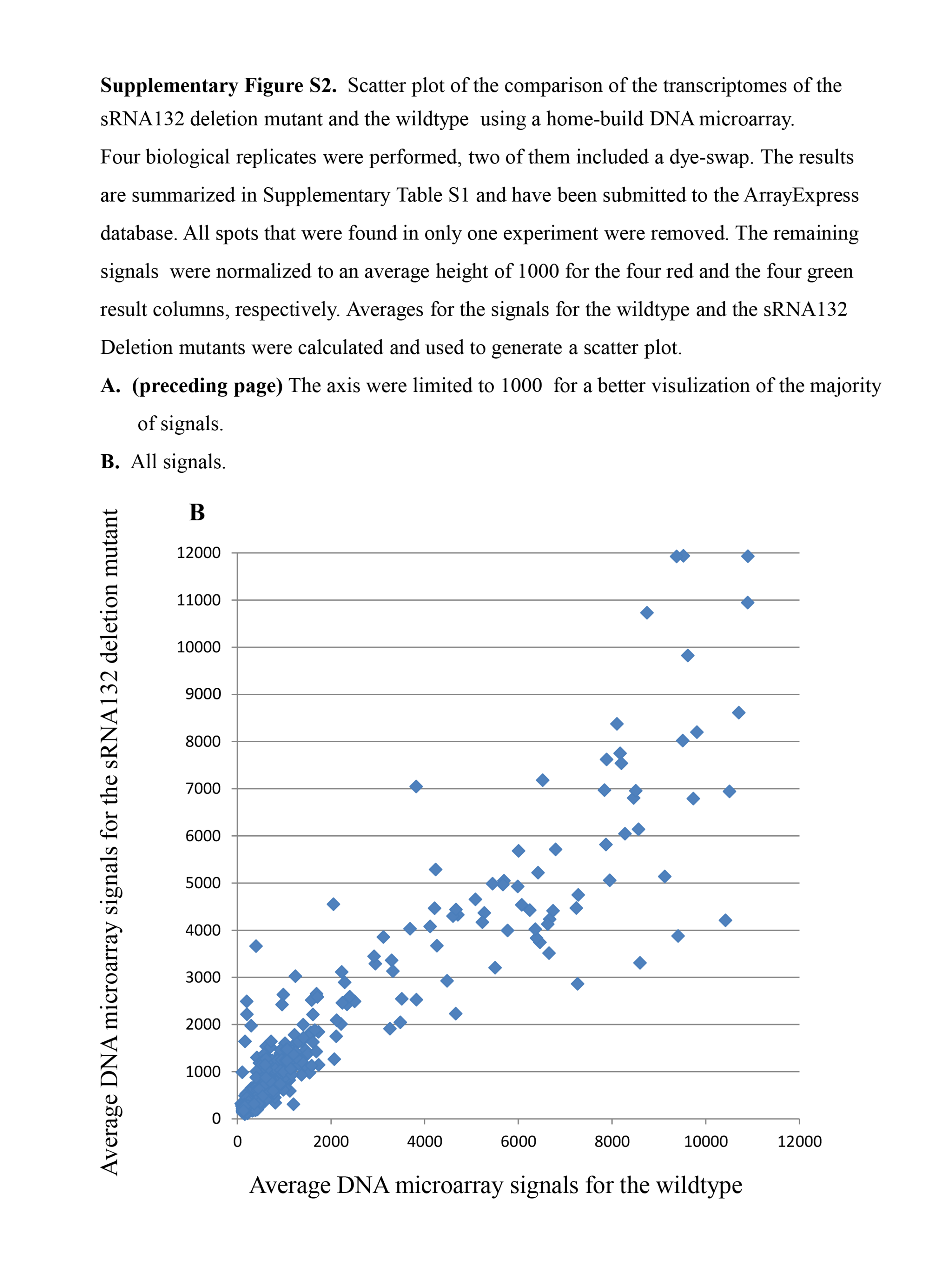

Supplement: Supplementary file 8 [file Image_3.TIF]
